# Performance of clay pigeon shooters in multiple object tracking under different target loads: skill level and gender differences

**DOI:** 10.3389/fpsyg.2026.1879857

**Published:** 2026-08-11

**Authors:** Kai Jin, Pingze Gao, Xupeng Zhang

**Affiliations:** 1 Department of Physical Education, Southeast University, Nanjing, Jiangsu, China; 2 Liaoning Provincial Sports Bureau, Shenyang, China; 3 Department of Physical Education, Nanjing University of Aeronautics and Astronautics, Nanjing, China

**Keywords:** attentional resources, clay pigeon shooting, cognitive expertise, gender differences, multiple object tracking

## Abstract

**Objective:**

To examine the effect of target load on the multiple object tracking performance of clay pigeon shooters, and to investigate differences in skill level (professional vs. amateur) and gender in this context. The attentional patterns in clay pigeon shooting differ fundamentally from those in ball sports. It remains unclear whether expert advantages exist in multiple object tracking among clay pigeon shooters, and whether long-term training can eliminate gender-based cognitive differences.

**Methods:**

Forty clay pigeon shooters (20 professionals, 20 amateurs; 20 males, 20 females) completed a multiple object tracking task with target loads of 2, 3, 4, 5, and 6. The dependent variable was tracking accuracy. A 2 (group) × 5 (target load) repeated-measures ANOVA was conducted, followed by simple effects analysis. Gender differences were examined using a separate 2 (gender) × 5 (target load) ANOVA.

**Results:**

Tracking accuracy decreased as target load increased (main effect of target load: *ηp*^2^ = 0.793). No differences were found between professional and amateur shooters when tracking 2 or 3 targets. However, under target loads of 4, 5, and 6, professionals showed higher tracking accuracy than amateurs (group × target load interaction: *ηp*^2^ = 0.095; simple effects: *p* = 0.003, 0.005, and 0.014, respectively). No differences were observed between male and female athletes under any target load condition (main effect of gender: *ηp*^2^ = 0.055, *p* = 0.145; gender × target load interaction: *ηp*^2^ = 0.024, *p* = 0.431).

**Conclusion:**

Clay pigeon shooters exhibit an expert advantage under high target loads in multiple object tracking tasks. This advantage may reflect domain-general cognitive resources such as working memory updating, attentional reorienting, and inhibitory control, rather than sport-specific multiple-object attentional capacity. No performance differences were observed between male and female athletes, suggesting that long-term intensive training may eliminate the gender gap in spatial attention commonly found in the general population. High-load multiple object tracking tasks may serve as a cognitive diagnostic tool for athlete selection in clay pigeon shooting.

## 1 Introduction

Clay pigeon shooting is the only shooting discipline that involves shooting at high-speed moving aerial targets (Causer et al., 2010; Gao et al., 2025). During competition, the clay target flies out at a maximum speed of 110 km/h, and athletes must complete the entire sequence of technical actions—from tracking the target, raising the gun, swinging the gun, to pulling the trigger—within a very short time after the target is launched (Abernethy and Neal, 1999; International Shooting Sport Federation, 2022; Morrillo et al., 2006). This process relies entirely on the efficient integration of the visual nervous system and shooting techniques (Causer et al., 2011). Therefore, dynamic visual attention ability is considered a key cognitive factor influencing performance in clay pigeon shooting (Liu et al., 2024; Lu et al., 2021).

The ability of dynamic visual attention refers to the capacity to continuously track target objects in a dynamic environment, persistently reallocate attentional resources across the temporal dimension, and simultaneously process multiple spatial locations (Huff et al., 2010; Meyerhoff et al., 2017). In sports, the visual information on the field is intricate and constantly changing, requiring athletes to fully utilize their dynamic visual attention abilities in such dynamic environments. They must employ the most optimal attentional allocation strategies to process complex visual information (Gou and Li, 2025b; Liu et al., 2024). In clay pigeon shooting, athletes not only need to focus on the clay target itself but also simultaneously process environmental information such as weather, lighting, and wind speed, which places extremely high demands on the allocation of attentional resources (Caroux et al., 2015; Gong et al., 2025; Shi et al., 2023).

In the study of dynamic visual attention abilities, the multiple object tracking (MOT) paradigm proposed by Pylyshyn and Storm (1988). has become a classic tool for investigating the processing mechanisms of visual attention in dynamic scenarios (Meyerhoff et al., 2017). This paradigm requires participants to simultaneously track several designated targets (typically 2–6) among a set of randomly moving identical objects, and to report the positions of the targets after the motion ceases. The MOT task effectively reflects an individual’s attentional resource capacity, working memory ability, and information processing speed in dynamic scenes (Liu et al., 2024; Styrkowiec et al., 2026; Tullo et al., 2024). A large body of research has shown that MOT performance is highly correlated with individuals’ attentional control abilities, and significant differences exist across different populations (e.g., athletes vs. non-athletes, experts vs. novices) (Mackenzie et al., 2024; Marion et al., 2025; Tullo et al., 2024).

Although the MOT paradigm has been widely applied in the field of sport psychology, a systematic review of the existing literature reveals that current research on multiple object tracking, both domestically and internationally, has primarily focused on ball sports such as basketball, soccer, table tennis, and volleyball (Gao et al., 2025; Zheng et al., 2024). These studies have shown that ball sport athletes exhibit faster visual information processing speeds and higher tracking accuracy in MOT tasks compared to ordinary college students. However, there are fundamental differences in visual attention patterns between clay pigeon shooting and ball sports: ball sport athletes need to simultaneously track the movements of multiple players and the ball in complex dynamic scenes, whereas clay pigeon shooters need to maintain continuous tracking of a single, small, high-speed moving target (Harris et al., 2020; Hüttermann et al., 2014; Nascimento et al., 2021; Qiu et al., 2018). This difference means that the research conclusions drawn from ball sports cannot be directly generalized to clay pigeon shooting.

Among ball sport athletes, evidence has shown that professional athletes exhibit superior tracking performance compared to novices when the number of targets increases (e.g., from 4 to 6), reflecting the pattern that “the higher the cognitive load, the more pronounced the expert advantage”(Gou and Li, 2025a; Guo and Wang, 2025; Qiu et al., 2019). However, whether such an advantage exists in clay pigeon shooting remains unknown. The reason this cannot be directly inferred from ball sports is that there are three fundamental differences in visual attention patterns between the two: 1. Ball sport athletes, during daily training and competition, need to simultaneously attend to the movements of multiple teammates, opponents, and the ball, thereby accumulating extensive experience in allocating attention across multiple targets. In contrast, clay pigeon shooters typically need to track only a single clay target during daily training and rarely need to allocate attention to multiple targets simultaneously (Causer et al., 2011; Hüttermann et al., 2019). 2. Ball sports require distributed attention, simultaneously focusing on multiple positions and multiple sources of information on the field; whereas clay pigeon shooting requires focused attention, maintaining continuous tracking of a single high-speed target while suppressing environmental distractions. These two attentional modes may rely on different cognitive processing mechanisms (Causer et al., 2011; Nebel et al., 2005). 3. In ball sports, the trajectory of the ball is relatively predictable (constrained by physical laws such as gravity and bounce), and athletes can anticipate its path based on opponents’ body postures. In contrast, in clay pigeon shooting, the direction and angle of the clay target’s launch are random, and its flight speed is extremely fast, imposing different demands on visual attention processing (Memmert, 2009). Therefore, the research conclusions regarding the expert advantage in MOT from ball sports cannot be directly extrapolated. Whether clay pigeon shooters exhibit a similar high-load expert advantage in multiple object tracking tasks still requires direct empirical validation.

**Figure 1:**
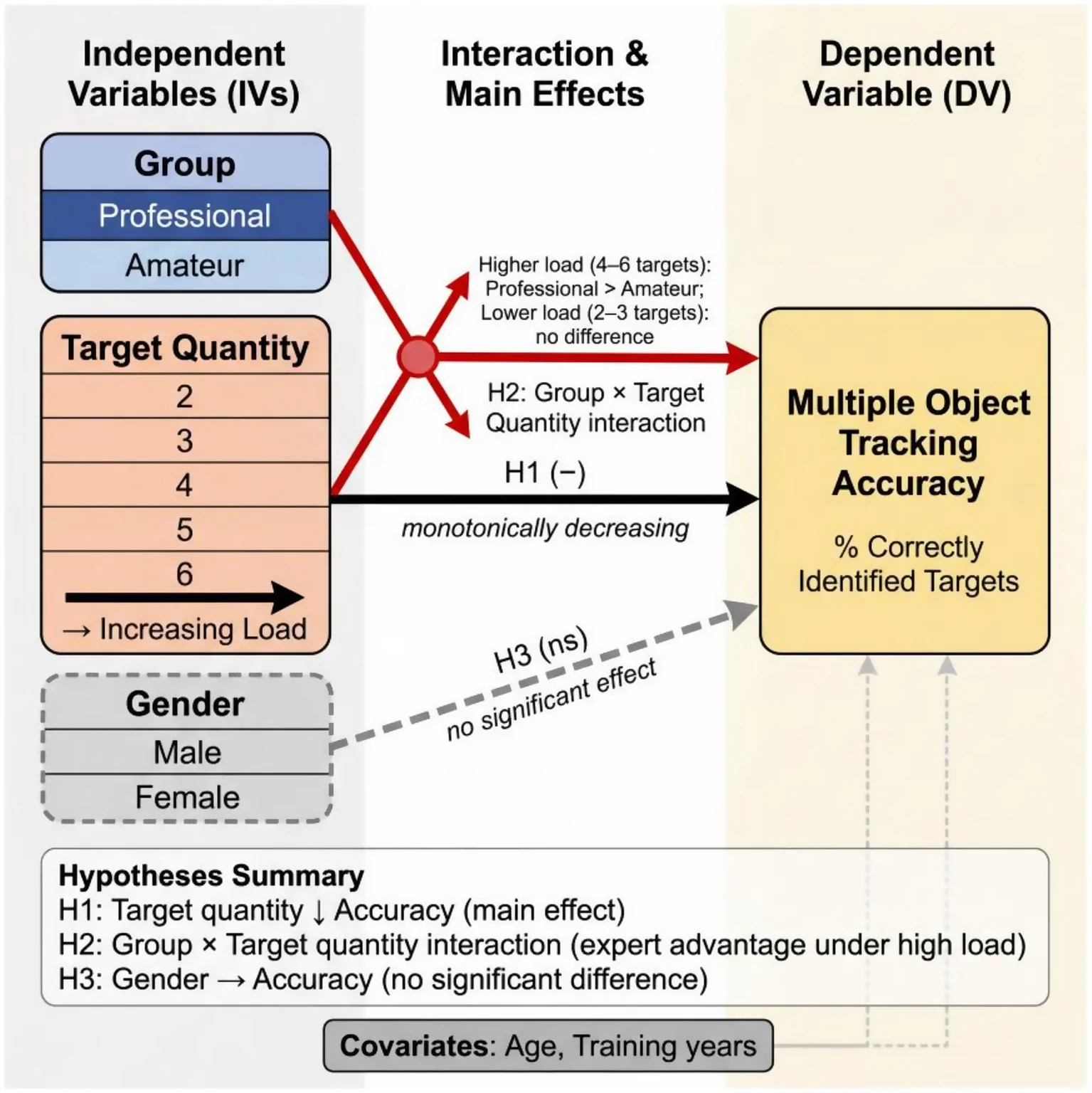
Conceptual model diagram of the hypothesized relationships among group, target load, gender, and multiple object tracking accuracy.

In the general population, males typically outperform females in MOT tasks, which has been attributed to innate advantages in spatial attention abilities among males (Feng et al., 2007; Voyer et al., 1995). However, some studies suggest that long-term sport-specific training may reduce or even eliminate this gender gap in cognitive abilities (Voss et al., 2010; Voyer and Jansen, 2017). In clay pigeon shooting, male and female athletes compete against each other under the same rules, which provides a unique research context for examining whether training can eliminate gender differences in cognition. However, no study to date has directly compared the performance of male and female clay pigeon shooters on MOT tasks (see Figure 1).

Based on the above, we propose the following three hypotheses:

*H1*: Tracking accuracy will decrease monotonically as target load increases.

*H2*: Under low load (2–3 targets), there will be no significant difference between the professional and amateur groups; however, under high load (4–6 targets), the professional group will perform significantly better than the amateur group.

*H3*: There will be no significant differences between male and female athletes under any target load condition.

## 2 Methods

### 2.1 Research design

A one-factor repeated-measures mixed design was employed to examine performance differences in a multiple object tracking task among clay pigeon shooters of different skill levels. The independent variables were group (a between-subjects variable with two levels: professional and amateur) and target load (a within-subjects variable with five levels: 2, 3, 4, 5, and 6). The dependent variable was tracking accuracy (the percentage of correctly identified target balls relative to the total number of target balls that should have been tracked).

### 2.2 Participants

#### 2.2.1 Sample size estimation

Using G*Power 3.1.9.2 software, based on a repeated-measures ANOVA (5 levels for the within-subjects factor, 2 levels for the between-subjects factor, *α* = 0.05, statistical power 1−*β* = 0.80, medium effect size *ηp*^2^ = 0.06, and correlation among repeated measures *r* = 0.5), the estimated minimum required sample size was 34 participants. Considering a 20% attrition rate, 50 participants were recruited, resulting in a final valid sample of 40 participants. The choice of a medium effect size (*ηp*^2^ = 0.06) was based on Cohen’s (1988) conventional benchmarks and is consistent with the meta-analytic effect sizes reported for athlete versus non-athlete comparisons in MOT tasks (Liu et al., 2024). As no prior MOT study exists specifically for clay pigeon shooters, a medium effect size was adopted as a conservative estimate.

#### 2.2.2 Inclusion and exclusion criteria

Through convenience sampling, clay pigeon shooters were recruited from the Fangshan Training Base Provincial Team in Jiangsu Province and the Shenyang Military Sports Land-Based Sports School. Inclusion criteria were as follows: ① Engaged in specialized training in skeet or trap shooting for ≥ 2 years; ② Binocular best-corrected distance visual acuity (LogMAR) ≤ 0.1 (decimal visual acuity ≥ 0.8), measured using the ETDRS chart at 4 meters; ③ Stereoacuity ≤ 60 arcseconds (Titmus stereotest); ④ No manifest strabismus (negative cover test); ⑤ Right-handedness (confirmed by the Annett Hand Preference Questionnaire). Exclusion criteria were: ① Presence of color blindness or color deficiency (Ishihara color vision test); ② History of ocular surgery or eye injury within the past 3 months; ③ Currently taking medications known to affect attention (e.g., sedatives, antidepressants); ④ Inability to complete the experimental tasks (e.g., comprehension difficulties).

#### 2.2.3 Group classification criteria

Participants were assigned to the professional or amateur group according to the following operational criteria.

##### 2.2.3.1 Professional group—all of the following conditions were met

① National first-tier athlete certification or above, issued by the General Administration of Sport of China (including national master-level athletes; a national champion title or medal at the National Shooting Championships was required for master-level classification);

② Specialized training in skeet or trap shooting for ≥ 6 years;

③ Weekly training volume ≥ 30 h;

④ Active participation in provincial-level or above competitions within the preceding 12 months.

##### 2.2.3.2 Amateur group—all of the following conditions were met

① National second-tier athlete certification in skeet or trap shooting;

② Specialized training in skeet or trap shooting for ≥ 2 but < 6 years;

③ Weekly training volume ≥ 30 h (matched to the professional group to control for current training intensity);

④ No participation in national-level competitions within the preceding 12 months.

#### 2.2.4 Final sample

The final sample included 40 athletes, divided into two groups: Professional group (*n* = 20): National first-tier athletes or above, including 4 master-level athletes and 16 first-tier athletes. Mean age = 19.91 ± 2.85 years, mean years of training = 6.77 ± 1.59 years, mean weekly training = 34 ± 2 h. There were 10 males and 10 females. Amateur group (*n* = 20): National second-tier athletes. Mean age = 16.33 ± 1.97 years, mean years of training = 4.74 ± 1.08 years, mean weekly training = 34 ± 2 h. There were 10 males and 10 females.

There were significant differences between the two groups in age (*t* = 4.46, *p* < 0.001) and years of training (*t* = 4.74, *p* < 0.001), with the professional group being higher, which was consistent with the expectation of stratification by skill level. No difference was found in gender distribution between the two groups (*χ*^2^ = 0.00, *p* = 1.00). Informed consent was obtained from all participants, and for minors, consent was also obtained from their legal guardians. The study was approved by the Ethics Committee of Southeast University (Approval No.: SEU-IRB-2025-12).

### 2.3 Experimental equipment and materials

The experimental program was written using Matlab R2016a (The MathWorks, Inc., 2005) and Psychtoolbox-3 (Brainard, 1997). Stimuli were presented on a Lenovo Air14ALC2021 laptop with a 14-inch screen, resolution of 1920 × 1,080 pixels, vertical refresh rate of 60 Hz, and average luminance of 150 cd/m^2^. The screen was placed on an adjustable lift table, and participants fixed their head position using a chin rest, with a viewing distance of 60 cm (the chin rest was aligned with the screen center). The response device was a wired USB mouse (Logitech G102, sampling rate 125 Hz). Ambient illumination in the laboratory was maintained at a constant 300 lx (measured with a TES-1335 illuminance meter).

The stimuli consisted of 12 white circles (RGB: 255, 255, 255; diameter: 1.2 cm, approximately 1.15° of visual angle), uniformly distributed within the central area of the screen (range: horizontal ±12°, vertical ±9°). The background was black (RGB: 0, 0, 0). During the indexing phase, the target circles turned blue (RGB: 0, 0, 255) and flashed three times (200 ms on, 200 ms off per flash). During the tracking phase, all circles moved randomly and independently at a constant speed of 5°/second. The motion model was “gravity-free linear rebound”; upon colliding with the boundaries, they followed the law of reflection (angle of incidence equals angle of reflection). No collision responses were programmed between circles (overlap and occlusion were allowed). The motion stopped after 10 s.

### 2.4 Experimental procedure

#### 2.4.1 Pre-experimental preparation

Participants were prohibited from consuming caffeinated beverages or engaging in strenuous exercise within 24 h prior to the experiment. Upon entering the laboratory, they first underwent 5 min of dark adaptation (using an eye mask). Subsequently, visual function screening was conducted (see criteria in Section 2.2.2). After passing the screening, participants sat in front of the computer and adjusted the chin rest to a comfortable position. The experimenter confirmed that the participant could clearly see the central fixation cross (“+”) on the screen. Then, eight practice trials were administered (the practice trials used a fixed target load of 3 and a speed of 5°/s, with the same content as the formal experiment) to ensure that participants understood the task requirements. Data from the practice trials were not included in the formal analysis.

#### 2.4.2 Formal experiment

The formal experiment consisted of five blocks, corresponding to tracking 2, 3, 4, 5, and 6 targets, respectively. Each block contained 30 trials. The order of the blocks was counterbalanced across participants using a Latin square design to eliminate order effects. A one-minute rest interval was given between blocks. The entire experiment lasted approximately 28 min, and no time pressure instructions (i.e., no requirement to respond as quickly as possible) were given.

**Figure 2:**
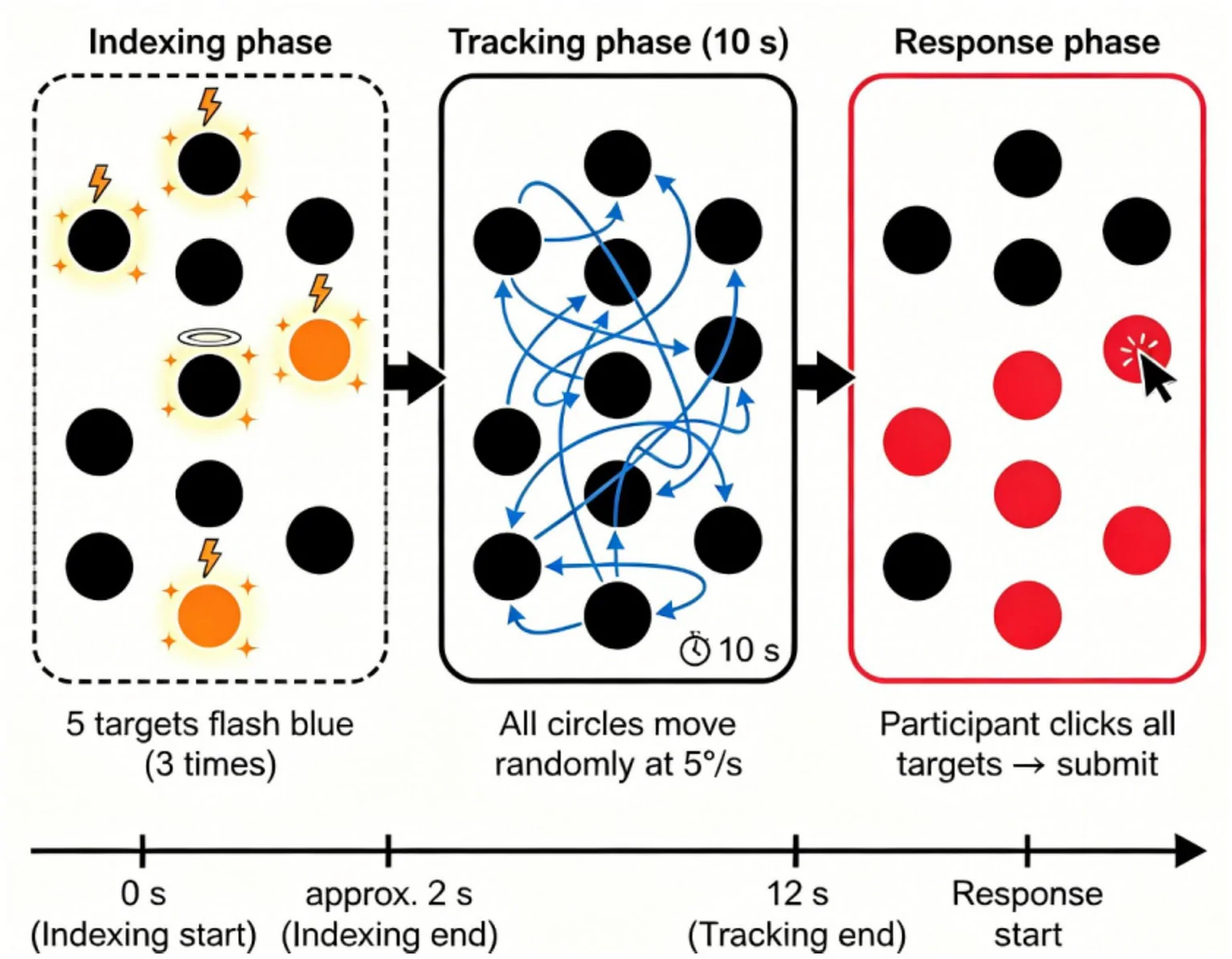
Examples of the multiple object tracking process under different target loads.

The procedure for each trial (using 5 targets as an example, see Figure 2):

A white fixation cross (“+”) was presented at the center of the screen for 1,000 ms.

Twelve white circles were presented for 500 ms.

Indexing phase: Among the circles, five turned blue and flashed three times (200 ms each flash), marking them as target circles; the remaining seven remained white and served as non-target circles.

All circles turned back to white for 200 ms.

Tracking phase: All circles moved randomly and independently at a speed of 5°/s for 10 s.

The motion stopped, and a prompt text appeared at the center of the screen: “Please use the left mouse button to click on all the target balls you were supposed to track.” Participants then clicked on all the balls they believed were the targets. Each clicked ball turned green (visual feedback). Participants could change their selections (deselect and reselect) and finally clicked a “Confirm” button to submit their response.

After submission, the screen was cleared for 500 ms, and the next trial began.

In each trial, the initial spatial positions of the target and non-target circles during the indexing phase were pseudo-randomly generated, with the constraint that the Euclidean distance between any two target circles was ≥ 2 cm (approximately 1.9° of visual angle) to avoid initial overlap. The seed values for the trajectories during the tracking phase were randomized to ensure non-repetition across different trials for the same participant.

#### 2.4.3 Data recording

The Matlab program automatically recorded the following variables for each trial: participant ID, group, target load condition, trial number, the IDs of the circles clicked by the participant (in the order of clicking), the number of correctly identified target circles, the number of false alarms (non-target circles clicked), and the total number of targets to be tracked. Raw data were saved in CSV format. Accuracy was calculated as: (number of correctly identified target circles ÷ total number of target circles to be tracked) × 100%. If a participant clicked on a non-target circle, no penalty was applied (it only affected the calculation of correct hits), but the number of false alarms was separately recorded for exploratory analysis.

### 2.5 Data processing and statistical analysis

#### 2.5.1 Data cleaning

Trials meeting any of the following criteria were excluded: ① Reaction time <100 ms or >5,000 ms; ② The participant’s head visibly left the chin rest during the tracking phase. The proportion of excluded trials was less than 3%, and the exclusion rate was balanced across conditions. No imputation for missing values was performed. Of the 40 participants, 32 (80%) had at least one trial excluded. The number of excluded trials per participant ranged from 0 to 14 (M = 4.2, SD = 3.6). No participant had all trials excluded in any condition. The proportion of excluded trials did not differ significantly between groups (professional vs. amateur, *p* = 0.62) or across target load conditions (*p* = 0.41).

#### 2.5.2 Statistical software and hypothesis testing

Statistical analyses were conducted using SPSS 26.0 (IBM Corp., Armonk, NY, USA) and JASP 0.17 (for Bayesian validation). A repeated-measures ANOVA was employed:

Model 1: The dependent variable was accuracy. The between-subjects factor was group (professional, amateur), and the within-subjects factor was target load (2, 3, 4, 5, 6).

Model 2 (exploratory): The between-subjects factor was gender (male, female), and the within-subjects factor was target load. Model 3 (ANCOVA, sensitivity analysis): A linear mixed model was conducted with group, target load, and their interaction as fixed effects, age as a continuous covariate, and participant as a random intercept, to test whether the group × target load interaction remained significant after controlling for age differences. Model 4 (exploratory, three-way ANOVA): A 2 (gender) × 2 (group) × 5 (target load) linear mixed model was conducted to test whether any gender effects depended on skill level.

The sphericity assumption was tested using Mauchly’s test. If violated, the Greenhouse–Geisser correction was applied when *ε* < 0.75, and the Huynh-Feldt correction was applied when ε > 0.75. *Post hoc* multiple comparisons were performed using Bonferroni correction. Simple effects analyses were conducted when interactions were significant, also using Bonferroni correction.

#### 2.5.3 Effect sizes and statistical power

Partial eta squared (*ηp*^2^) was reported as the effect size measure, with values of 0.01, 0.06, and 0.14 interpreted as small, medium, and large effects, respectively. Post hoc power analyses based on the obtained sample (G*Power, repeated-measures ANOVA, within-subjects correlation *r* = 0.5, *α* = 0.05, observed *ηp*^2^) showed that for the main effect of target load (expected *ηp*^2^ ≈ 0.15), power exceeded 0.99; for the group × target load interaction effect (expected *ηp*^2^ ≈ 0.08), power was 0.81. All tests were two-tailed, with the significance threshold set at *α* = 0.05.

## 3 Result

### 3.1 Descriptive statistics

The multiple object tracking accuracy (M ± SD) of participants in each group under different target load conditions is presented in Table 1 (professional vs. amateur) and Table 2 (male vs. female). Overall, as the number of tracking targets increased from 2 to 6, accuracy showed a decreasing trend across all participants.

**Table 1:** Tracking accuracy between groups under different target load conditions (M ± SD).

Group	2 targets	3 targets	4 targets	5 targets	6 targets
Professional group	0.965 ± 0.06	0.860 ± 0.13	0.770 ± 0.13	0.620 ± 0.18	0.410 ± 0.23
Amateur group	0.945 ± 0.06	0.850 ± 0.11	0.605 ± 0.19	0.445 ± 0.18	0.255 ± 0.14

**Table 2:** Tracking accuracy between genders under different target load conditions (M ± SD).

Gender	2 targets	3 targets	4 targets	5 targets	6 targets
Male	0.963 ± 0.05	0.900 ± 0.08	0.691 ± 0.16	0.573 ± 0.19	0.350 ± 0.22
Female	0.945 ± 0.07	0.800 ± 0.12	0.683 ± 0.21	0.483 ± 0.20	0.333 ± 0.20

### 3.2 Main effect of target load on tracking accuracy

Repeated-measures ANOVA (Table 3) revealed a significant main effect of target load (*F* = 145.86, *p* < 0.001, *ηp*^2^ = 0.793), indicating that tracking accuracy decreased significantly as target load increased. Post hoc comparisons (Bonferroni correction, see Table 4) further confirmed that accuracy for 2 targets was significantly higher than for 3 targets (*p* < 0.001), accuracy for 3 targets was significantly higher than for 4 targets (*p* < 0.001), accuracy for 4 targets was significantly higher than for 5 targets (*p* < 0.001), and accuracy for 5 targets was significantly higher than for 6 targets (*p* < 0.001).

**Table 3:** Results of repeated-measures ANOVA for tracking accuracy between two groups under different target loads.

Main effects and interaction effects	Type III sum of squares	Degrees of freedom	*F*	Significance	*ηp* ^2^
Target load	9.941	3.262	145.86	<0.001	0.793
Group × target load	0.272	3.262	3.998	0.008	0.095
Group	0.551	1	11.745	0.001	0.236

**Table 4:** Post hoc pairwise comparisons of tracking accuracy across target loads (Bonferroni corrected).

(I) Target load	(J) Target load	Mean difference (I−J)	Standard error	Significance
2	3	0.100	0.021	0.000
	4	0.267	0.026	0.000
	5	0.422	0.029	0.000
	6	0.623	0.030	0.000
3	2	−0.100	0.021	0.000
	4	0.167	0.028	0.000
	5	0.322	0.029	0.000
	6	0.523	0.034	0.000
4	2	−0.267	0.026	0.000
	3	−0.167	0.028	0.000
	5	0.155	0.032	0.000
	6	0.355	0.034	0.000
5	2	−0.422	0.029	0.000
	3	−0.322	0.029	0.000
	4	−0.155	0.032	0.000
	6	0.200	0.026	0.000
6	2	−0.622	0.030	0.000
	3	−0.522	0.034	0.000
	4	−0.355	0.034	0.000
	5	−0.200	0.026	0.000

### 3.3 Group differences and interaction effects

The main effect of group was significant (*F* = 11.745, *p* = 0.001, *ηp*^2^ = 0.236), indicating that the professional group had significantly higher overall tracking accuracy than the amateur group.

The group × target load interaction effect was significant (*F* = 3.998, *p* = 0.008, *ηp*^2^ = 0.095). Simple effects analysis (Table 5) showed that:

**Table 5:** Pairwise comparisons between groups under different target loads.

Target load	(I) Group	(J) Group	Mean difference (I−J)	Standard error	Significance	95% CI lower bound	95% CI upper bound
2	Professional	Amateur	0.020	0.019	0.295	−0.018	0.058
3	Professional	Amateur	0.010	0.037	0.788	−0.065	0.085
4	Professional	Amateur	0.165	0.053	0.003	0.059	0.271
5	Professional	Amateur	0.175	0.058	0.005	0.057	0.293
6	Professional	Amateur	0.155	0.060	0.014	0.033	0.277

Under target loads of 2 and 3, there was no significant difference in accuracy between the professional and amateur groups (*p* > 0.05).

Under a target load of 4, the professional group (0.770 ± 0.13) had significantly higher accuracy than the amateur group (0.605 ± 0.19), *p* = 0.003.

Under a target load of 5, the professional group (0.620 ± 0.18) had significantly higher accuracy than the amateur group (0.445 ± 0.18), *p* = 0.005.

Under a target load of 6, the professional group (0.410 ± 0.23) had significantly higher accuracy than the amateur group (0.255 ± 0.14), *p* = 0.014.

**Figure 3:**
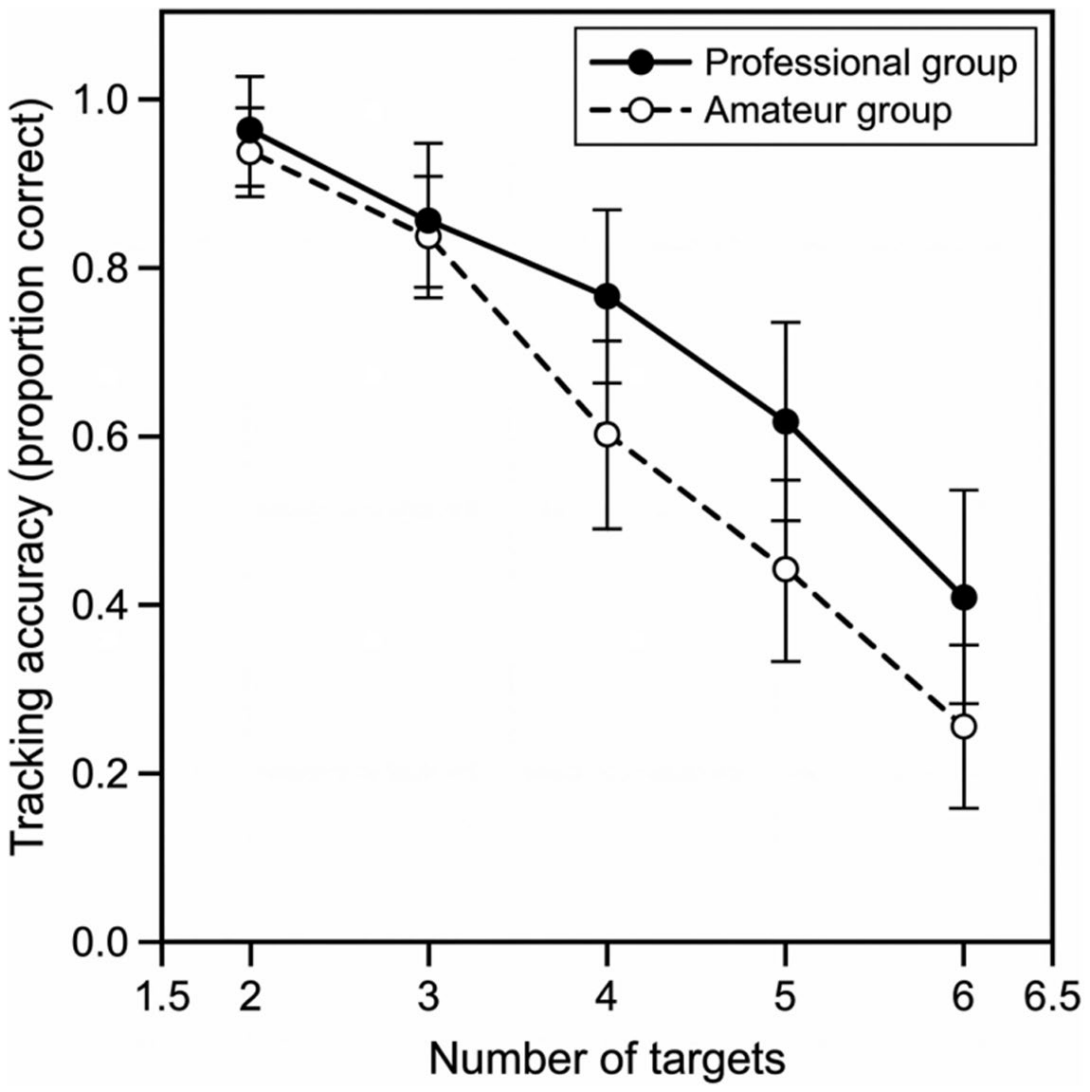
Comparison of tracking accuracy between the two groups under different target loads.

Figure 3 visually illustrates the trends in accuracy changes for the professional and amateur groups under different target loads. The two curves nearly overlap at target loads of 2 and 3, while the professional group shows notably higher accuracy than the amateur group at target loads of 4, 5, and 6.

#### 3.3.1 Controlling for age

To test the robustness of the group × target load interaction after controlling for age differences between the professional and amateur groups, a linear mixed model was conducted with group, target load, and their interaction as fixed effects, age as a continuous covariate, and participant as a random intercept. The results showed that age was not a significant predictor of tracking accuracy [Wald *χ*^2^(1) = 0.127, *p* = 0.721]. After controlling for age, the group × target load interaction remained significant [Wald *χ*^2^(4) = 15.992, *p* = 0.003]. The pattern of simple effects was consistent with the primary analysis: no significant group difference at target loads of 2 or 3 (*p* > 0.05), but the professional group significantly outperformed the amateur group at target loads of 4 (*p* = 0.013), 5 (*p* = 0.008), and 6 (*p* = 0.021). The main effect of group was attenuated after controlling for age [Wald *χ*^2^(1) = 0.265, *p* = 0.607], suggesting that the overall group difference in accuracy partly reflects age-related variance between the professional and amateur samples. Critically, however, the persistence of the group × target load interaction indicates that the expertise effect under high cognitive load cannot be attributed solely to age differences. These findings confirm that the expert advantage observed under high target loads (4–6) is robust to age adjustment. The results of the primary ANOVA and the ANCOVA sensitivity analysis are therefore consistent in supporting H2.

### 3.4 Gender difference analysis

In this analysis, male and female participants were collapsed across professional and amateur groups (*n* = 20 per gender), consistent with the equal distribution of gender across groups (10 males and 10 females in each group). A repeated-measures ANOVA with gender as the between-subjects variable (see Table 6).

**Table 6:** Results of repeated-measures ANOVA for tracking accuracy between genders under different target loads.

Main effects and interaction effects	Type III sum of squares	Degrees of freedom	*F*	Significance	*ηp* ^2^
Target load	9.851	3.146	133.972	<0.001	0.779
Gender × target load	0.069	3.146	0.932	0.431	0.024
Gender	0.129	1	2.219	0.145	0.055

The main effect of target load was significant (*F* = 133.972, *p* < 0.001, *ηp*^2^ = 0.779), consistent with the results reported above.

*Post hoc* comparisons (Bonferroni correction) across target load levels are presented in Table 7.

**Table 7:** *Post hoc* pairwise comparisons of tracking accuracy across target loads in the Gender Model (Bonferroni corrected).

(T) Target Load	(J) Target Load	Mean difference (T−J)	Standard error	Significance
2	3	0.104	0.021	0.000
	4	0.267	0.029	0.000
	5	0.426	0.031	0.000
	6	0.623	0.032	0.000
3	2	−0.104	0.021	0.000
	4	0.163	0.030	0.000
	5	0.322	0.032	0.000
	6	0.519	0.036	0.000
4	2	−0.267	0.029	0.000
	3	−0.163	0.030	0.000
	5	0.159	0.032	0.000
	6	0.357	0.034	0.000
5	2	−0.426	0.031	0.000
	3	−0.322	0.032	0.000
	4	−0.159	0.032	0.000
	6	0.197	0.026	0.000
6	2	−0.623	0.032	0.000
	3	−0.519	0.036	0.000
	4	−0.357	0.034	0.000

The main effect of gender was not significant (*F* = 2.219, *p* = 0.145, *ηp*^2^ = 0.055), indicating that there was no statistically significant difference in multiple object tracking accuracy between male and female athletes.

**Figure 4:**
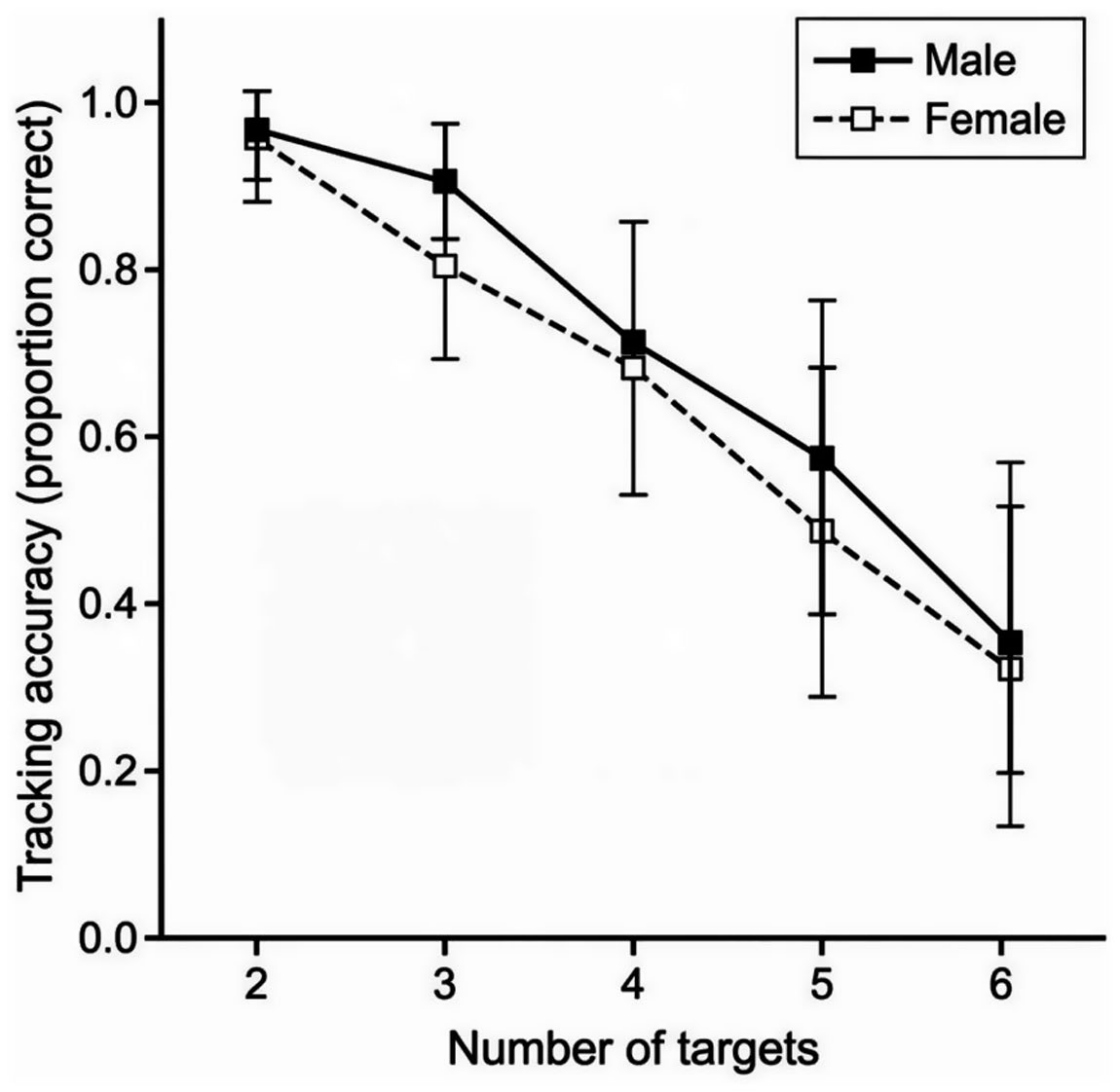
Interaction trend between gender and target load.

The gender × target load interaction effect was not significant (*F* = 0.932, *p* = 0.431, *ηp*^2^ = 0.024), indicating that the decline in accuracy with increasing target load followed a similar pattern for male and female athletes (see Table 2, Figure 4).

Figure 4 shows that the accuracy of both male and female athletes decreased synchronously as target load increased, with the two curves being approximately parallel, further supporting the conclusion that the interaction effect was not significant.

As an exploratory analysis, a 2 (gender) × 2 (group) × 5 (target load) linear mixed model was conducted to test whether gender effects depended on skill level. The gender × group interaction was not significant [Wald *χ*^2^(1) = 0.187, *p* = 0.666], indicating no overall gender difference by skill level. The gender × group × target load three-way interaction was significant [Wald *χ*^2^(4) = 10.649, *p* = 0.031]. However, simple interaction analyses at each target load (gender × group, uncorrected for multiple comparisons) revealed that this effect was primarily driven by the 6-target condition (gender × group at 6 targets: *p* = 0.015), where the direction of the gender difference reversed across groups: professional males (M = 0.510) outperformed professional females (M = 0.310), whereas amateur females (M = 0.300) outperformed amateur males (M = 0.210). Given the small cell sizes (*n* = 10 per subgroup) and the absence of correction for multiple comparisons, this three-way interaction should be interpreted with caution and warrants replication in larger samples. Across all other target loads (2–5), gender × group interactions were non-significant (all *p* > 0.05). Overall, these results provide partial support for H3: the absence of a gender main effect and the non-significant gender differences at target loads 2–5 are consistent with the hypothesis that long-term training eliminates broad gender gaps in spatial attention. However, the significant three-way interaction at the 6-target load suggests that H3’s strong version (no gender differences under any condition) may not hold at extreme cognitive loads, where subtle gender-by-expertise interactions emerge. Given the small cell sizes (n = 10 per subgroup), this finding should be interpreted cautiously and warrants replication before firm conclusions are drawn.

## 4 Discussion

This study is the first to systematically examine performance differences in multiple object tracking (MOT) tasks among clay pigeon shooters of different skill levels, while also testing the role of gender. The main findings are as follows: (1) Tracking accuracy decreased monotonically as target load increased, supporting H1; (2) No significant difference was found between the professional and amateur groups under low load (2–3 targets), but the professional group performed significantly better than the amateur group under high load (4–6 targets), supporting H2; (3) No significant differences were observed between male and female athletes under any target load condition, and there was no gender × target load interaction, supporting H3.

Consistent with H1, tracking accuracy significantly decreased for all participants as the target load increased from 2 to 6 (*ηp*^2^ = 0.793, large effect). This finding is highly consistent with the classic MOT literature and reflects the limited capacity of the human dynamic visual attention system. When the number of targets to be tracked simultaneously exceeds approximately 3–4, the allocation of attentional resources gradually shifts from parallel processing to time-sharing of resources (Alvarez and Franconeri, 2007; Holcombe and Chen, 2013). For clay pigeon shooters, although they rarely need to track multiple clay targets simultaneously during sport-specific training, the present results suggest that they are still subject to the hard constraints of basic cognitive capacity when faced with an overload of dynamic visual information. Notably, even under the 6-target condition, the mean accuracy of the professional group was only about 41%, and that of the amateur group was about 25%. This relatively low accuracy—even among professional shooters—may partly reflect the fundamental difference in attentional demands between the two types of sport: ball sport athletes are regularly exposed to dynamic multi-target scenarios during training and competition, which may foster relatively efficient multiple object tracking strategies, whereas the attentional system of clay pigeon shooters is predominantly adapted to sustained focusing and high-speed tracking of a single target. However, as the present study did not include a ball-sport control group and task parameters (target load, speed, tracking duration) vary across published studies, direct cross-sport performance comparisons should be made with caution. Qualitatively, this pattern aligns with the fundamental difference in attentional modes between the two types of sport: clay pigeon shooting demands sustained focused attention on a single high-speed target, whereas ball sports require distributed attention across multiple players and the ball. The low accuracy of clay pigeon shooters under high target loads therefore does not reflect a cognitive deficit but rather the mismatch between the task’s attentional demands and the attentional strategies honed through sport-specific training (Brinkbäumer et al., 2024; Heilmann et al., 2022).

The absence of a group difference under low target loads (2–3 targets) warrants explicit discussion. At two targets, tracking accuracy approached ceiling for both groups (professional: 96.5%; amateur: 94.5%), leaving minimal room for between-group differentiation—a classic ceiling effect (Uttl, 2005). At three targets, the cognitive demand remains within the typical capacity limit of the human attentional tracking system. Classic MOT studies have established that untrained individuals can reliably track approximately 3–4 objects simultaneously (Pylyshyn and Storm, 1988; Alvarez and Franconeri, 2007; Meyerhoff et al., 2017). Thus, tracking three targets does not yet exceed the baseline attentional resources available to amateur shooters, and the professional group’s cognitive reserve is not recruited.

The group difference emerged only when the target load surpassed this capacity boundary (≥4 targets), at which point the amateur group’s performance dropped steeply (from 85% at 3 targets to 60.5% at 4 targets), while the professional group was able to partially compensate (from 86% at 3 targets to 77% at 4 targets), drawing on domain-general cognitive resources strengthened through years of intensive training. This pattern suggests that the professional group’s advantage is not a general superiority across all task conditions but rather a high-load-specific cognitive reserve—a finding consistent with the broader expertise literature demonstrating that expert–novice differences are magnified under conditions of increased cognitive demand (Memmert, 2009; Voss et al., 2010). Importantly, good MOT accuracy at low target loads does not necessarily translate to superior shooting performance, because low-load conditions do not sufficiently tax the cognitive resources that are shared between MOT and sport-specific demands. It is the ability to maintain tracking accuracy under high cognitive load—where working memory updating, attentional reorienting, and inhibitory control are heavily engaged—that is more likely to differentiate experts from amateurs and potentially to relate to real-world shooting performance.

The core finding of this study is that the difference in MOT accuracy between the professional and amateur groups emerged only when the target load was ≥ 4 (interaction effect *ηp*^2^ = 0.095, medium effect). This pattern is similar to the “cognitive load dependence of expert–novice differences” observed in ball sports (Memmert, 2009), but the underlying mechanisms may be quite different. In ball sports, the expert advantage is typically attributed to domain-specific knowledge structures and optimized attentional allocation strategies (Gorman et al., 2013; Newberry et al., 2021). However, clay pigeon shooters almost never engage in multiple object tracking tasks during their daily training. Therefore, it is unlikely that they have accumulated domain-specific knowledge of “multi-target attentional allocation” through sport-specific training (Oppici et al., 2017; Prenzel and Mandl, 1993). Then, where does the professional group’s advantage under high load come from?

We propose a novel explanatory pathway: high-load MOT tasks may recruit domain-general cognitive resources that are shared with the specialized abilities required in clay pigeon shooting, rather than relying solely on multi-target attentional allocation per se. Specifically: 1. Working memory updating: When tracking 6 targets, participants must continuously refresh the most recent position of each target, which relies on the central executive system of working memory. Clay pigeon shooters need to continuously update the orientation, velocity, and angle information of the clay target within an extremely short period during high-speed flight. This “dynamic position updating” ability may be strengthened through long-term specialized training. 2. Attentional reorienting: In MOT tasks, when occlusion or overlap occurs among targets, attention must rapidly reorient to the occluded targets. In clay pigeon shooting, the clay target may briefly pass through background shadows or areas with changing illumination, requiring shooters to similarly “recapture” the target quickly. 3. Inhibitory control: High-load MOT requires individuals to suppress interference from non-target circles. Clay pigeon shooters need to suppress environmental distractions (wind speed, lighting, audience noise) as well as their own emotional fluctuations during competition. This domain-general inhibitory control ability may transfer to the MOT task. It is important to emphasize, however, that this explanatory pathway remains speculative, as the present study did not include direct measures of the proposed mediating cognitive processes. The claim that working memory updating, attentional reorienting, and inhibitory control mediate the expert advantage in high-load MOT is inferred from the shared task demands of clay pigeon shooting rather than empirically demonstrated. Future research should adopt a multi-task design that includes established cognitive paradigms—such as the N-back task for working memory updating, the Posner cueing paradigm for attentional reorienting, and the Flanker task for inhibitory control—to formally test the proposed mediation pathway.

Applying the multiple object tracking paradigm to clay pigeon shooting has revealed that the expert advantage pattern in this sport is superficially similar to that in ball sports, but the underlying mechanisms are different. Performance of clay pigeon shooters on MOT tasks does not reflect their sport-specific multi-target attentional ability (as there is little demand for multi-target attention in the sport itself), but rather reflects domain-general cognitive abilities such as working memory updating, attentional reorienting, and inhibitory control (Allen et al., 2006; Lapierre et al., 2017). Therefore, high-load multiple object tracking tasks can serve as tools to probe these foundational cognitive resources. This also suggests that researchers should select cognitive paradigms based on the attentional mode of the sport (distributed vs. focused, multi-target vs. single-target) and avoid inappropriate cross-sport generalizations.

There was no significant main effect of gender on MOT accuracy, nor was there a significant gender × target load interaction. This result directly supports H3. In the general population, a large body of research has reported that males outperform females on MOT tasks (Jin et al., 2022; Legault and Faubert, 2024), typically attributed to innate male advantages in spatial rotation, mental rotation, and attentional allocation (Brown, 2013; Enge et al., 2023). However, in the present study, the performance of male and female athletes largely overlapped. How can this “disappearance of the gender gap” be explained? We propose that the most plausible mechanism is the shaping effect of long-term, high-intensity sport-specific training on cognitive functions (Yan et al., 2025; Zhang et al., 2025). Clay pigeon shooting is a sport that places extremely high demands on dynamic visual attention, hand-eye coordination, and decision-making under pressure. Male and female athletes compete against each other under the same rules, with identical daily training time and training content (target tracking, gun raising, gun swinging, triggering), and both achieve a weekly training volume of 34 ± 2 h. This high-intensity, highly consistent, long-duration training may have produced two effects: (1) through sport-specific training, female athletes have enhanced their visual attention, working memory updating, and inhibitory control abilities to a level comparable to that of male athletes, thereby compensating for any innate spatial attention disadvantages that may exist (Lucia et al., 2023); (2) female athletes who persist in clay pigeon shooting and reach the national second-tier level or above may already be in the high percentile of the female population in terms of dynamic visual attention ability, reflecting a combined effect of self-selection and training-based selection (de Amorim et al., 2024).

It is worth noting that this study is not the first to report that sports training can eliminate gender differences in cognition. In sports such as basketball and fencing, expert female athletes have been shown to perform at levels comparable to, or even better than, their male counterparts on certain visual cognitive tasks (Jin et al., 2023). Our results extend this phenomenon to clay pigeon shooting and confirm it for the first time using the MOT paradigm. This provides new empirical evidence for the hypothesis that “long-term sports training can eliminate innate cognitive gender differences” and suggests that the underlying mechanisms may involve a combination of training-driven cognitive compensation and selective sampling. This finding has important implications for understanding the relative contributions of “nature and nurture” in the development of cognitive abilities. It should be noted that the effect size for the main effect of gender was only *ηp*^2^ = 0.055 (small to medium), with *p* = 0.145. Future studies with larger sample sizes and Bayesian factor analyses are needed to quantify the strength of evidence supporting the null hypothesis.

Integrating the above three findings yields a more systematic theoretical picture. First, the high-load expert advantage exhibited by clay pigeon shooters in the MOT task does not arise from sport-specific training in multi-target attentional allocation, but rather because high-load MOT taps into the foundational cognitive resources that are strengthened by this sport: working memory updating, attentional reorienting, and inhibitory control. This implies that even for seemingly unrelated cognitive tasks, expert advantages may emerge as long as the basic cognitive processes they recruit overlap with those trained in the sport. Second, this advantage only becomes apparent after the cognitive load exceeds a threshold, suggesting that when assessing athletes’ cognitive abilities, sufficiently challenging task parameters must be used; otherwise, floor or ceiling effects may mask true between-group differences. Finally, the absence of gender differences in MOT performance among athletes further supports the view that long-term, high-intensity training can reshape the distribution of cognitive abilities, and also reminds researchers that gender effects observed in athletic populations cannot be directly equated with gender differences in the general population.

It is important to clarify that MOT accuracy per se is not a direct proxy for shooting performance. The present study did not collect competition scores or on-field shooting accuracy data, and therefore cannot establish a direct predictive relationship between MOT performance and on-field achievement. The rationale for proposing high-load MOT as a cognitive diagnostic tool rests on the inference that the domain-general cognitive resources taxed by the MOT task (working memory updating, attentional reorienting, inhibitory control) overlap with those required during actual clay pigeon shooting—not on the assumption that multi-object tracking is itself a sport-specific skill (Mann et al., 2007; Voss et al., 2010).

Previous meta-analyses have shown that while expert athletes generally outperform novices on domain-general cognitive measures, the effect sizes are typically larger for sport-specific paradigms than for generic laboratory tasks (Mann et al., 2007; Voyer and Jansen, 2017). Therefore, establishing the empirical association between MOT performance and shooting competition outcomes—ideally through prospective longitudinal designs in which baseline high-load MOT performance predicts subsequent improvements in competition hit rates—is a necessary next step before MOT-based athlete selection tools can be recommended with confidence. Future studies should incorporate competition scores (e.g., hit rate across qualification and final rounds), shooting accuracy indices, and multi-season tracking to directly test the predictive validity of high-load MOT for clay pigeon shooting performance.

Based on the above findings, we propose the following training and selection recommendations: First, high-load MOT tasks (e.g., 4–6 targets) can serve as cognitive diagnostic tools for assessing working memory updating and inhibitory control abilities in clay pigeon shooters, and future efforts may explore incorporating them into athlete selection testing. Second, for amateur athletes, progressive MOT training (gradually transitioning from 3 to 5 targets) could be introduced to enhance their attentional allocation and updating abilities under high cognitive load. Although the sport itself does not directly demand multi-target attention, improvements in foundational cognitive resources may indirectly facilitate sport-specific performance (e.g., maintaining attentional focus more stably when rapidly tracking a single clay target). Third, given that no significant gender differences in MOT performance were observed, coaches should not presume an “innate female disadvantage” in dynamic visual attention. Cognitive training programs can be designed equally for male and female athletes without gender-specific adjustments.

The present study has the following main limitations: First, the sample size was relatively small (20 per group), and the non-significant main effect of gender may be limited by statistical power. Future studies should expand the sample and incorporate Bayesian factor analyses. Second, there were significant differences in age and years of training between the professional and amateur groups. Although this is consistent with stratification by skill level, age itself may be related to cognitive development. Future research could employ matched-sample designs or analysis of covariance to control for age. Third, the MOT task was a laboratory paradigm, which has a gap in ecological validity compared to real clay pigeon shooting scenarios. Future studies could use virtual reality or portable eye-tracking to enhance ecological validity. Fourth, the present study did not directly measure mediating variables (e.g., working memory updating, inhibitory control), so the interpretation of “foundational cognitive resources” remains speculative. Future research should incorporate tasks such as N-back and Flanker for mediation analyses. Fifth, a control group from the general population was lacking, making it impossible to definitively determine whether the “disappearance of gender differences” is due to training or to sample selection bias. Future studies should include a control group of ordinary college students for comparison. Sixth, participants were drawn from both skeet and trap shooting disciplines, which differ in target distance and trajectory; discipline-specific effects on MOT performance could not be examined due to limited subgroup sample sizes. Seventh, male and female shooters compete in separate Olympic events, and the exploratory three-way interaction at the 6-target load suggests that discipline-specific gender effects may exist, warranting replication in larger, discipline-stratified samples.

## 5 Conclusion

This study found that: 1.multiple object tracking accuracy decreases as target load increases; 2.professional clay pigeon shooters outperform amateurs only under high load (4–6 targets), reflecting enhanced domain-general cognitive resources (working memory updating, attentional reorienting, and inhibitory control) rather than sport-specific multi-target attention; 3 no gender differences exist in tracking accuracy, suggesting that long-term high-intensity training may eliminate the typical spatial attention gender gap. High-load MOT tasks may serve as a cognitive diagnostic tool for athlete selection in clay pigeon shooting.

## Data Availability

The original contributions presented in the study are included in the article/supplementary material, further inquiries can be directed to the corresponding author.
